# Myasthenia gravis in Latin America and the Caribbean: epidemiology, resources, and accessibility to diagnosis and treatment

**DOI:** 10.3389/fpubh.2026.1791605

**Published:** 2026-04-08

**Authors:** Valeria L. Salutto, Cristian E. Navarro, Alejandra Urra Pincheira, David R. Dondis Camaño, Karina Guerrero, André C. J. dos Santos, Carlos A. Moreno Martinez, Víctor D. Ojeda Jacquet, Lenin Peñaloza Miranda, Abayubá Perna Ramos, Guillermo Romero Suárez, Edwin S. Vargas Cañas, Juan Francisco Idiaquez Rios, Carolina Barnett-Tapia

**Affiliations:** 1 Medical Research Institute Alfredo Lanari, Buenos Aires University, Buenos Aires, Argentina; 2 School of Medicine and School of Economic Sciences, Universidad de Antioquia, Medellín, Colombia; 3 Neuromuscular Unit, Department of Neurology and Psychiatry, Clínica Alemana, Santiago, Chile; 4 Centro de Neurociencias, Ciudad de la Salud, Caja de Seguro Social, Ciudad de Panamá, Panama; 5 Universidad Central de Venezuela, Caracas, Venezuela; 6 Ribeirão Preto School of Medicine, University of São Paulo, São Paulo, Brazil; 7 Hospital del Valle, San Pedro Sula, Honduras; 8 Hospital Central IPS, Asunción, Paraguay; 9 Caja Nacional de Salud, La Paz, Bolivia; 10 Centro Privado Montevideo, Montevideo, Uruguay; 11 Hospital Metropolitano de Quito, Quito, Ecuador; 12 National Institute of Neurology and Neurosurgery, Neuromuscular Diseases Clinic, Mexico City, Mexico; 13 Division of Neurology, Department of Medicine, University of Toronto, Toronto, ON, Canada

**Keywords:** accessibility, health care costs, health economics, Latin America, myasthenia gravis

## Abstract

Latin-American and Caribbean (LAC) countries face persistent challenges in achieving universal health coverage, with out-of-pocket healthcare expenditures posing a significant barrier. Access to care for rare diseases, such as myasthenia gravis (MG), is significantly impacted. We conducted an exploratory descriptive study between LAC countries, and we assessed the availability of diagnostic and therapeutic resources for MG across 12 LAC countries, along with associated costs and access barriers. We found marked inequalities regarding the availability and coverage of diagnostic tests and therapies by public healthcare systems. The elevated out-of-pocket costs, up to several times the minimum wage, and shortage of neurologists, makes appropriate diagnosis and treatments inaccessible for many patients living with MG. Novel treatments for MG are unavailable for most people with MG in Latin America.

## 1 Introduction

Latin America and The Caribbean (LAC) encompass 33 low- and middle-income countries with an inequality gap and a rapidly aging population. An increasingly growing middle class that is better informed adds pressure to the fragmented health systems that strive to provide universal coverage (1). With a combined population exceeding 690 million, 80% residing in urban areas and 21% in informal settlements or inadequate houses, LAC faces unique challenges in delivering equitable healthcare (1). High levels of informality and disparities in access to basic services exacerbate these challenges, often surpassing those observed in other parts of the world (1, 2). There is marked segmentation and variability in healthcare systems across the region (3). This contributes to heterogeneous levels of health coverage in LAC countries, ranging from approximately 76% in high-coverage nations such as Argentina, Brazil, Colombia, Mexico, and Uruguay, to significantly lower rates in countries like Bolivia (4, 5).

Health systems include the public model, with public finance and provision; social security, including a national social security system; and the private one, consisting of voluntary insurance (6). These models coexist with variable hegemony. Sanitary indicators are dissimilar among the different countries, and some are outdated (5, 7, 8). The economy is developing, and the systems cannot guarantee proper and quality availability of technologies and medications (9). Main access barriers are out-of-pocket expenses on health, well above the average of countries from the Organization for Economic Cooperation and Development (OECD) (10, 11). In this context, rare diseases such as myasthenia gravis (MG) are often deprioritized in national health agendas, which tend to focus on nutritional deficiencies, infectious diseases and chronic cardiovascular conditions (1).

MG is a chronic autoimmune neuromuscular disorder with a global mean prevalence of 173.3 (95% confidence interval [95%CI] 129.7–215.5) cases per million and a mean incidence of 15.7 (95%CI 11.5–19.9) cases per million person-years (12). In patients with rare diseases, the journey from the onset of symptoms to diagnosis (i.e., diagnostic odyssey) can take several years (average 4–5 years or longer) (13). Specifically, the diagnosis of MG can be challenging because fatigue, muscle weakness, and other symptoms are common to many diseases (14). The ASPIRE project identified barriers to timely diagnosis of patients with generalized MG, such as physicians confusing MG symptoms with women’s health problems, difficulty in accessing specialist care, and lack of knowledge of MG among health professionals (15).

The economic burden of MG is substantial, estimated at $5,567 USD monthly per patient in the US, rising to $17,220 USD in case of crisis (16). Annual out-of-pocket costs (average, $15,798 USD) are primarily caused by medications, the cost of diagnosis, and health insurance premiums (17). Although scientific advances and novel therapies have transformed MG management in high-income countries, access to diagnostic tools and treatments in LAC remains scarce and uneven. Epidemiologic studies of MG in the region are limited and heterogeneous, reflecting different methodologies, environments, and unequal access to specialized care (18). There are no data from LAC countries regarding the economic burden of MG, nor for the available resources for diagnosis and treatments. We aimed to identify current access barriers and deficiencies in MG care across LAC, including availability of neurologists, diagnostic tests, and treatments, and their associated costs.

## 2 Methods

An exploratory descriptive study was conducted, combining a literature review and institutional database analysis (indirect sources) with a structured survey of neurologists managing MG patients across 12 LAC countries (direct source).

### 2.1 Indirect data sources

A comprehensive literature review was performed covering the period from 2010 to 2023. Databases included MEDLINE (via Pubmed), LILACS, and grey literature as policy revision, articles, Health Ministry webpages, and regional organization web pages. Publications in English, Spanish, and Portuguese were considered. The following MeSH and DeCS terms were used: Myasthenia Gravis; Epidemiology; Latin America; Health Status Indicators; Universal Health Coverage; Healthcare Financing; Public Health Services Coverage; Private Health Services Coverage; Diagnostic Tests; Drug Therapy; Out-of-Pocket Health Expenditures; Gross Domestic Product; Monthly Salary and Neurologist availability. Health indicators such as life expectancy at birth in LAC and other OECD countries were extracted (10).

Indirect data were synthesized and categorized into three domains:

- Healthcare indicators: Life expectance at birth and Mortality rate (19).
- Economic indicators: Minimum monthly salary, Gross Domestic Product (GDP) for 2022 and the percentage of GDP allocated to health in the same year.
- Epidemiological data: Neurologist density per country and MG prevalence across LAC countries.

### 2.2 Direct data sources

A structured questionnaire was administered to 12 neurologists from 12 LAC countries (Argentina, Bolivia, Brazil, Chile, Colombia, Ecuador, Honduras, Mexico, Panama, Paraguay, Uruguay, and Venezuela). These neurologists were identified during the 2023 Latin-American Neuromuscular Society (SOLANE) meeting, conducted in Bogotá, Colombia. Eligibility criteria included prior clinical experience in the management of MG. Survey responses were categorized into three modules:

- Diagnostic tests: Availability and cost of repetitive nerve stimulation (RNS), single-fiber electromyography (SFEMG), thoracic computed tomography (CT), and antibody testing.
- MG Treatments: Availability and cost of conventional therapies (pyridostigmine, corticosteroids, non-steroidal immunosuppressants, intravenous immunoglobulin and plasma exchange) and novel MG treatments (e.g., biologics).
- Healthcare coverage: Classification of the systems providing MG diagnostic tests and MG treatments, public and private healthcare systems of each country or international referral, and the degree of financial coverage. Private system was defined to include prepaid medical care, insurance policies, and private office care.

Data collection occurred between August and December 2023 via structured forms completed by participating neurologists. A follow-up questionnaire was distributed in January 2024 to clarify and/or expand specific variables. Responses were grouped by country and healthcare coverage type. Diagnostic procedures and pharmacological treatments costs were retrieved from responses provided by neurologists and databases of national regulatory agencies in each surveyed country. Appendix A shows the questionnaire.

### 2.3 Statistical analysis

Descriptive statistics (absolute and relative frequencies, median, interquartile range [IQR], mean, standard deviation [SD]) were calculated for categorical and quantitative variables as appropriate. No inferential statistical tests were applied due to the exploratory nature of the study and the limited sample size.

All cost-related data were converted from the national currency of each country into US dollars with an exchange rate for the year 2023 (20). To assess affordability the ratio between the monthly cost of common MG drugs (pyridostigmine, prednisone and azathioprine) if paid out-of-pocket, in relation to the minimum wage was calculated. The ratio was performed considering daily doses as follows: pyridostigmine 60 mg × 3 tablets, prednisone 15 mg and azathioprine 100 mg. Data analysis was conducted used Microsoft Excel-Office 365 (Microsoft Corporation, Redmond, WA, USA), and R software version 4.3.2 (The R Foundation for Statistical Computing, Vienna, Austria).

This study was based on a review of the literature as well as a review of local and available databases. No individual data were collected and therefore this study did not require ethics approval nor informed consent.

## 3 Results

### 3.1 Indirect data sources

Mean life expectance at birth for people in the 12 LAC countries was 76 years (21), compared to 81 years for OECD countries (10). There was marked regional gap; for example, life expectancy at birth in Bolivia was 68.5 years, while in Chile it was 81.2 years (21). Mortality ranged from 181.9 per 100,000 population in Chile to 492 per 100,000 population in Bolivia (22). The average rate of all causes of death in the LAC region was about 50% greater than the average for countries from the OECD (10).

Related to economic variables, there was wide variability in the monthly minimum salary for the 12 included countries, ranging between $15 and $1,320 (mean $399 ± 323), as well as in the average GDP that ranged between $5,553,100,597 and $924,000,000,000 (mean $ 405,617,609,787 ± 618,879,000,785) (23). The average percentage of GDP allocated to health was 8.04% (SD 0.016; range 4.55–10.1%) (see Table 1) (23).

**Table 1:** Economic and epidemiological characteristics in Latin American countries included in this study.

Country	Minimum monthly salary	Gross Domestic Product (GDP) 2022	Percentage of the GDP allocated to health 2022 (%)	Number of neurologists (per 100,000 inhabitants)	Myasthenia gravis prevalence (per 100,000 inhabitants)	Reference
Argentina	$132	$5,553,100,597	9.86	2.9	36.71	(29, 34)
Bolivia	$300	$44,401,000,000	8.43	0.19	No data	(24)
Brazil	$1,320	$1,924,000,000,000	9.14	2.46	No data	(30)
Chile	$527	$308,812,607,775	10.1	3.27	8.36	(28, 35)
Colombia	$273	$360,179,535,840	7.69	1.15	14.3	(33, 36)
Ecuador	$250	$118,141,073,235	7.53	0.54	No data	(57)
Honduras	$417	$31,718,000,000	8.28	0.29	No data	(25)
México	$365	$1,414,101,000,000	5.72	1.53	No data	(32)
Panama	$315	$77,814,000,000	8.47	0.82	No data	(26)
Paraguay	$370	$41,722,000,000	7.74	0.58	No data	(58)
Uruguay	$500	$59,000,000,000	8.95	3.8	6.3	(27, 37)
Venezuela	$15	$482,360,000,000	4.55	1.69	No data	(31)

Adapted from World Bank database.

Cost is standardized to 2023 US dollar.

The number of neurologists per 100,000 population was lowest in Bolivia (0.19) (24), followed by Honduras (0.29) (25), and Panama (0.82) (26). The country with the greatest availability of neurologists was Uruguay (3.8) (27), followed by Chile (3.27) (28), Argentina (2.9) (29), Brazil (2.46) (30), Venezuela (1.69) (31), México (1.53) (32), and Colombia (1.15) (33). Bolivia and Paraguay do not have official sources or epidemiological studies describing the rate of neurologists per 100,000 inhabitants; in these cases, a calculation was made between the number of neurologists extracted from an official source and the population of the last census (see Table 1).

The prevalence rate MG in Argentina was 36.71 per 100,000 person-years (between 2006 and 2012) (34); the prevalence per 100,000 population was 8.36 in Chile (2018) (35), 14.3 in Colombia (2023) (36), and 6.3 in Uruguay (between 1960 and 1976) (37) (Table 1). We did not find published estimates for the other countries.

### 3.2 Direct data sources

Every country has electrophysiologic studies available. Bolivia and Paraguay do not have SFEMG but have RNS. Overall, RNS is available in 11 (92%) countries; this is only through private health services in Bolivia, Honduras and Paraguay, and in both the public and private sectors in the rest of the countries. In Venezuela, RNS is not available in the public system, and we could not ascertain whether it is available in the private sector. SFEMG is available in 9 (75%) countries: in Brazil, Chile, Colombia, and Uruguay, it is available in both public and private systems, while in Argentina, Ecuador, Honduras, Mexico, and Panama, it is only available through private coverage. In Venezuela, SFEMG is not available in the public system, and we could not ascertain whether it is available in the private sector. Chest tomography (CT) is available in every country, and in 11 (92%) it is available in both public and private health systems, while in Venezuela, it is only available in the public system.

**Figure 1:**
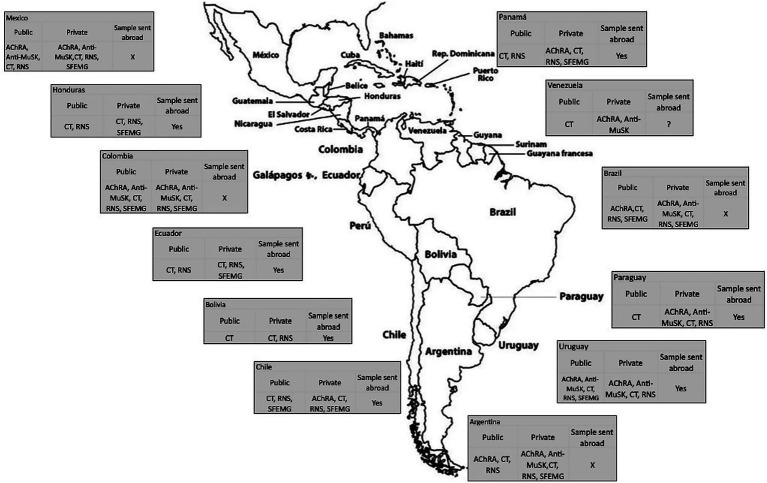
Coverage of diagnostic test for myasthenia gravis in 12 Latin American and Caribbean countries. This figure details the availability and coverage of diagnostic tests across public and private healthcare sectors for each country. “Sample sent abroad” indicates that the blood sample for antibody detection cannot be processed domestically. An “X” indicates that the sample is not forwarded to an international reference laboratory. A “?” denotes that no information is available. AChR, acetylcholine receptor; MuSK, muscle-specific kinase; CT, Chest CT; RNS, Repetitive nerve stimulation; SFEMG, Single fiber electromyography.

Acetylcholine receptor (AChR) antibody detection is done locally in 8 (67%) countries; this is available in the public and private systems in Argentina, Brazil, Colombia, Mexico and Uruguay; and only privately in Panama, Paraguay, and Venezuela. In Chile, Ecuador and Honduras, blood samples are sent to an international lab, processed by private healthcare system, and are paid by the patient. Muscle-specific tyrosine kinase (MuSK) antibody testing is done locally in 6 (50%) countries: in Colombia and Mexico, this test is available in both the public and private systems; in Argentina, Brazil, Paraguay and Venezuela it is only through the private system. In Chile, Ecuador, Honduras and Panama, blood samples are sent to a laboratory abroad and paid by the patient. In Uruguay, a private laboratory sends blood samples abroad for the anti-MuSK test; however, it is having a very high cost. In Bolivia, antibody tests are not available in the public system, and whether they are available through international laboratories via private healthcare is unknown. Table 2 summarizes the costs of different diagnostic tests, and Figure 1 shows coverage of common diagnostic test for MG per country.

**Table 2:** Costs of test used in the diagnosis of myasthenia gravis in 12 Latin American countries (prices in US dollars 2023).

Country	Anti-AChR		Anti-MuSK		Chest CT		Repetitive nerve stimulation		Single fiber electromyography	
	Public	Private	Public	Private	Public	Private	Public	Private	Public	Private
Argentina	$60	$100	Not available	$145	$65	$180	$16	$82	Not available	$170
Bolivia	No data	No data	Not available	No data	$217	$217	Not available	$22	Not available	Not available
Brazil	$19	$39	Not available	$145	$58	$97	$19	$97	$97	$194
Chile	Not available	$137*	Not available	$137*	$54	$54	$26	Variable	$57	$57
Colombia	$63	$63	$217	$217	$40	$80	$14	$28	$24	$48
Ecuador	Not available	$150*	Not available	$392*	$35	$60	$40	$60–140	Not available	$200
Honduras	Not available	$120*	Not available	$525*	$170	$170	Not available	$162	Not available	$303
Mexico	$15	$15–50	$180	$100–180	$100–380	$100–380	$50	$170–290	Not available	$780
Panama	Not available	$200	Not available	$120*	$50	$550	$50	$250	Not available	$250
Paraguay	Not available	$110	Not available	$110*	$50	$50	Not available	$65	No available	Not available
Uruguay	$250	$250	$1,500	$1500*	$100–200	$100–200	$125	$125	$190	$190
Venezuela	Not available	$25	Not available	$35	$5	No data	Not available	No data	Not available	No data

AChR, acetylcholine receptor; MuSK, muscle-specific kinase.

*Sample sent abroad.

Pyridostigmine is available in all countries; however, it is available exclusively through the private system in 5 (42%) countries: Bolivia, Ecuador, Honduras, Paraguay, and Venezuela. Pyridostigmine is available in both private and public systems in Argentina, Brazil, Chile, Colombia, Mexico, Panama, and Uruguay. Argentina has a national program that provides pyridostigmine free of charge for Argentine resident citizens through a bill for the MG Commemoration Day (*Ley 26903 del 13 de Noviembre del 2013* [bill 26903, November 13th, 2013]). Steroids are available in all countries, in 10 (83%) of them in the public system. Azathioprine is available in all the countries, 9 (75%) in the public system. In Argentina and Venezuela, access to steroids and azathioprine is only possible through the private system; in the remaining countries, they are available in the public and private sectors. Paraguay is the exception: both systems cover the cost of steroids, but the cost of azathioprine is only covered by the private system. Mycophenolate is available in all countries, in 8 (67%) through the public system. It is only available in the private system in Argentina, Honduras, Paraguay, and Venezuela. Access to tacrolimus is restricted through public health systems and is only offered in Brazil, Colombia and Mexico. No data are available for Bolivia and Paraguay. Tacrolimus is unavailable in Uruguay; the patient can access this medication through a judicial mechanism. In the remaining countries, it is only available in the private system.

Intravenous immunoglobulin (IVIg) is available in all countries but cannot be used as maintenance therapy for MG through any of the public healthcare systems. In the event of a myasthenic crisis one can use IVIg at the expense of public health system resources in Argentina, Bolivia, Brazil, Colombia, Mexico, Panama, Paraguay and Uruguay (67%). Plasmapheresis for MG crisis is available in Bolivia, Honduras, and Paraguay only through the private system. Colombia has plasmapheresis only in the public system. Rituximab is available (off-label) for MG through the public healthcare in Chile, Colombia, Mexico, Panama, Paraguay and Uruguay.

Novel treatments are overall inaccessible. Only in the private system an individual with refractory MG can access eculizumab treatment in Argentina, this through legal recourse. Ravulizumab is approved in Argentina, Brazil, Chile, Ecuador, Mexico and Panama, but not covered; patients can have access to ravulizumab if they file a legal recourse (according to the legislation of each country). In the rest of the countries, the drugs are not available. Table 3 depicts the coverage of common MG medications.

**Table 3:** Coverage of myasthenia gravis treatments in 12 Latin American countries.

Drug	Public system coverage only	Private system coverage only	Public and Private system coverage
Pyridostigmine	7 (58%)	5 (42%)	12 (100%)
Prednisone	10 (83%)	2 (17%)	12 (100%)
Azathioprine	9 (75%)	3 (25%)	12 (100%)
Mycophenolate	8 (67%)	4 (33%)	12 (100%)
Tacrolimus	3 (25%)	7 (58%)	10 (83%)
Rituximab	6 (50%)	6 (50%)	12 (100%)
Cyclophosphamide	7 (58%)	2 (17%)	9 (75%)
IVIG (crisis)	8 (67%)	4 (33%)	12 (100%)
Plasma Exchange	9 (75%)	2 (17%)	11 (92%)
Complement C5 inhibitor	0 (0%)	0 (0%)	0 (0%)

Table 4 presents the costs of common MG medications. The monthly cost of MG treatments, if paying out-of-pocket, as a percentage of the minimum wage is variable. Considering a standard daily treatment of prednisone 15 mg, azathioprine 100 mg, and pyridostigmine 180 mg (60 mg three times a day), an individual would pay as low as 6% of the minimum wage in Brazil, 15% in Chile, 170% in Bolivia and up to 423,000% in Venezuela. Overall, in 6 (50%) of the countries a month of MG treatment has out-of-pocket costs >50% of the minimum wage (Supplementary Table 1).

**Table 4:** Cost of myasthenia gravis treatments in 12 Latin American countries.

Country	Pyridostigmine 60 mg tablet	Pyridostigmine monthly	Prednisone 5 mg tablet	Prednisone monthly	Azathioprine 50 mg tablet	Azathioprine monthly
Argentina	$0.50	$45	$0.24	$21.60	$0.50	$30.00
Bolivia	$1.00	$90	$2.00	$180.00	$4.00	$240.00
Brazil	$0.16	$14.40	$0.38	$34.20	$0.40	$24.00
Chile	$ 0.74	$66.6	$0.03	$2.70	$0.17	$10.20
Colombia	$2.21	$198.9	$0.61	$54.90	$0.54	$32.40
Ecuador	$0.50	$45	$0.12	$10.80	$0.78	$46.80
Honduras	$2.00	$180	$0.12	$10.80	$1.50	$90.00
México	$0.90	$81	$0.14	$12.60	$3.00	$180.00
Panama	$0.60	$54	$0.30	$27.00	$0.60	$36.00
Paraguay	$0.55	$49.5	$0.20	$18.00	$0.80	$48.00
Uruguay	$1.88	$169.2	$0.04	$3.60	$0.32	$19.20
Venezuela	$0.65	$58.5	$30.00	$2,700.00	$60.00	$3,600.00

Cost is standardized to 2023 US dollar.

Monthly cost considering the following doses: Pyridostigmine 60 mg three times per day, Prednisone 15 mg per day and Azathioprine 100 mg per day. Calculation of the monthly cost according to the price of each medication in each country.

## 4 Discussion

This study provides an overview of the availability of diagnostic and therapeutic resources for MG in LAC countries. The results highlight substantial disparities among countries in access to health technologies, ranging from those considered essential for the care of MG patients to more advanced interventions, particularly regarding treatments such as monoclonal antibodies. This reflects the described differences in health system structures and the variability in the availability of trained personnel for the management of these patients. Our analysis identified marked heterogeneity in health indicators, such as life expectancy and mortality rate, emphasizing the profound inequities in the region’s health systems (10). Importantly, our data suggest that a higher GDP (global or per capita), does not necessarily translate into better access to health technologies within public health systems or private insurance coverage.

Chile is the country that assigns the most resources as GDP percentage (10.1%), followed by Argentina (9.86%) and Brazil (9.14%). Venezuela has the lowest resource allocation, with 4.55% of the GDP. It is worth noting that in low- and middle-income countries, optimal financial mechanisms to achieve Universal Health Coverage remain an ongoing subject of debate, and there is no universally accepted guideline for tax-based health financing beyond the general recommendation of allocating approximately 5% of GDP to healthcare (38).

Access to neurologists is the first barrier to a prompt diagnosis of MG, and while this was variable within LAC countries, all were below the mean of 7.1 neurologists per 100,000 inhabitants reported for high income countries, and below the WHO recommendation (5 neurologists per 100,000 inhabitants) (39, 40). In our survey, Uruguay had the highest density of neurologists (3.8/100,000 inhabitants), whereas Bolivia had only 0.19 per 100,000 inhabitants. A lack of neurology specialists in Latin America, may be largely due to very limited institutional opportunities of training throughout the region, for example, only 0.67% of the residency vacancies in Argentina in 2023 were assigned to neurology (41). For many LAC professionals seeking training in neurology, the only option is to go abroad. Furthermore, accessing a specialized program in neuroimmunology or neuromuscular medicine is even more challenging (40). There are no data regarding the number of neurologists with neuromuscular training in LAC countries, but very few countries have subspecialty programs and therefore this number is likely very low, and concentrated in large cities, increasing disparities for patients in smaller cities and rural areas (29, 42).

Delays in accessing neurological consultations for diagnosis may be attributed to a shortage of specialists, geographic barriers requiring long-distance travel for patients in nonurban LAC settings, and a lack of urgent referrals from primary care physicians. Preliminary data from a multicenter retrospective study in Argentina reported a mean diagnostic delay—defined as the interval between symptom onset and MG diagnosis—of 422.6 days (SD 771.5 days), with significantly shorter delays among patients initially evaluated by neurologists compared with those first seen by other specialists (mean 116.5 days vs. 459.1 days; *p* = 0.015) (43). Comparatively a multicenter European study including five countries reported a mean diagnostic delay of 363.1 days (SD 520.9 days) (44). Diagnostic errors may also contribute to delayed recognition of MG; misdiagnosis as a psychiatric disorder has been associated with presentation as a myasthenic crisis before the correct diagnosis is established (45).

These findings are consistent with data from the ASPIRE project, which described the complexity of the diagnostic journey for patients with generalized MG. The study showed that racial and ethnic minority groups, as well as female patients, experienced longer and more burdensome diagnostic pathways (15). A delay in MG diagnosis would be associated with a delay in initiating appropriate treatment and a higher risk of myasthenic exacerbations/crises and comorbidities. Evidence suggests that patients with longer diagnostic delay may experience higher disease burden, comorbid anxiety and depression, greater involvement of healthcare professionals, and lower health-related quality of life than those with early diagnosis (44).

The variability in MG prevalence across the LAC region may be attributed to methodological discrepancies or diagnostic inaccuracies—including misdiagnosis, underdiagnosis, and overdiagnosis—which likely reflect unequal access to specialized neurological care. For instance, the prevalence reported in Argentina (36.71 per 100,000) significantly exceeds the global mean of 17.33 per 100,000 estimated in a recent systematic review (12). Furthermore, it is four times higher than the prevalence reported in Chile (8.36 per 100,000). This striking disparity can largely be explained by differences in study design: the Argentine estimate derives from a retrospective study at a single private referral hospital in Buenos Aires (34), whereas the Chilean study utilized a capture–recapture methodology, cross-referencing a national pyridostigmine registry with a patient survey (35). Another crucial factor is the global rise in very late-onset MG (patients over 65 years of age), a demographic that frequently remains underdiagnosed (18, 46, 47). Additionally, regional heterogeneity may be driven by variations in environmental risk factors, ethnic diversity, and socioeconomic status. To the best of our knowledge, no Latin American genetic studies are currently available linking familial inheritance to disease prevalence, prognosis, and response to treatment. Finally, recent infectious outbreaks have introduced new epidemiological variables (48). While an Italian study may not establish a direct association between incident MG cases and COVID-19 infection or vaccination, data from Brazil indicated that the rise in MG-related mortality (from 0.76% in 2011 to 1.90% in 2023) was closely linked to COVID-19 infections among unvaccinated individuals (30).

Although a high proportion of the countries surveyed have the technologies required to diagnose MG, significant disparities exist, particularly regarding their low availability within public healthcare systems. For example, SFEMG, the most sensitive test (49), is only available in the public system in 33% of the 12 ascertained countries. While the prices we obtained do not reflect out-of-pocket costs, as some patients have insurance, many individuals without coverage would not be able to afford this. For example, the cost of SFEMG in Mexico can reach up to $780, almost two times the minimum wage. Similarly, AChR antibody testing is only available through the public system in 42% countries, and therefore, limited for those individuals of low income and/or without private insurance. Of note, we only ascertained the availability of commercial AChR antibody, and cell-based assays were not available at time of data collection. Anti-MuSK testing is even more limited, only publicly funded in 17% of countries and available through private systems with a high cost, up to $1,500 in Uruguay, three times the minimum wage. The need to send samples abroad for AChR and MuSK antibody analysis represents a critical barrier, exacerbating diagnostic delays and significantly increasing the cost of the process. All countries have CT scanners in the public system, but the numbers are below the OECD average, which is ~30 per 1,000,000 population; in our study even the countries with highest number of scanners (Brazil and Chile) were below the OECD mean (50). Significant disparities in diagnostic delay are also expected between patients in the public healthcare system versus those in the private sector (51).

Regarding treatments for MG, prednisone is available in all countries, and publicly funded in 83%, followed by 75% public coverage for azathioprine, and 67% public coverage for mycophenolate. Pyridostigmine—the only drug in the WHO list of essential medications for MG—is only covered publicly in 58% of the countries ascertained, with a high cost in some up to $2.21 for each tablet, for example in Colombia. While it is not a disease-modifying treatment, it is considered as the first line of treatment by guidelines from high income countries (52, 53). At the time of our data collection, of the novel treatments for MG, only eculizumab and ravulizumab had been approved in some LAC countries, but are available almost exclusively in the private sector, through complex administrative and legal procedures. Effectively, novel treatments are inaccessible for most people living with MG in LAC. The emergence of new high-cost therapies for MG increases will exacerbate health financing issues in LAC countries and will increase judicial procedures to ensure rights protection and healthcare access (17). Given that some countries have limited public funding for standard of care treatments for MG, such as pyridostigmine, it is unlikely that novel and expensive treatments will be accessible for most patients.

In LAC, the health system is segmented since most countries have subsystems with doubled functions of governance, financing, and service provision; this is the key to resource misuse (38). Public resources deficit gives rice to private intervention in individual insurance and service delivery (6). For individuals without coverage through the public healthcare system, out-of-pocket costs can be exceedingly high and limit access to diagnosis and standard of care treatments. Out-of-pocket health costs are one of the main obstacles to accessing care (10, 17, 54).

In 2019, health expenditure represented, on average, 6.9% of the GDP in LAC compared to 8.5% in OECD countries. In LAC countries, 60% of health expenditure comes from governmental and mandatory insurance plans, while out-of-pocket payments, voluntary payments, and external resources plans cover the remaining 40%. Conversely, in OECD countries, governmental and mandatory insurance represent 77% of the health expenditure. In LAC in 2019, on average, 32.4% of health expenditure was paid out of pocket, above the 20% average in OECD (55). The high level of out-of-pocket expenses in LAC is evidence of weak health systems and low coverage levels (1). In 2015, Argentina spent 10.2% of its GDP on health care but only 3% on public health, with a per-capita health expenditure of $1,390; however, Mexico and Brazil had similar public health expenditures (2.2–3.0% and 3.3–4.5%, respectively). In Argentina, 5% of the population expends more than 25% of the household expenditure on healthcare, while in Brazil it is 13% (4). The high dependency on out-of-pocket expenses produces a significant inequality in access to diagnosis and treatment, especially for rare diseases such as MG. In the past years there has been exponential increase in novel and more targeted treatments for MG; however, these are currently only accessible in high income countries and will likely remain inaccessible for most patients worldwide. Consequently, cost plays a critical role in decisions regarding treatment initiation (56).

### 4.1 Limitations

This study has several limitations. First, to ensure broad geographic representation, data collection relied on a single key informant per country. Although these participants were highly experienced neuromuscular specialists with deep knowledge of local diagnostic pathways and healthcare barriers, relying on a single expert may not fully capture the intra-national variability of each healthcare system. Nevertheless, this approach facilitated standardized data collection and cross-country comparisons. A second limitation is the scarcity of standardized data regarding available health technologies and costs in several countries. The high number of private providers across the region drives significant cost variability, limiting our analysis to the best available estimates rather than exact figures. However, our primary objective was to highlight regional disparities in healthcare access rather than to conduct a formal economic evaluation, which would require a different methodological design. Despite its exploratory nature, this study successfully identifies critical gaps and inequalities in the management of MG across LAC. These findings should be interpreted with caution and serve as a foundational step for future multicenter studies involving a greater number of specialists to validate and expand upon these regional nuances.

## 5 Conclusion

The study shows significant disparities between countries in terms of health indicators and healthcare financing. This variability is also observed in the management of MG, as evidenced by the scarcity of epidemiological studies, low number of neurologists, the lack of specialized tests for MG diagnosis, and even their complete absence in some countries. Most diagnostic tests are characterized by limited availability, inadequate public coverage, and significant cost variability. Regarding treatment, while conventional therapy is widely available, disparities exist in terms of cost and coverage. Access to biologic medications is generally obtained through legal procedures.

Despite the availability of diagnostic tests and standard treatments for MG in LAC, inequalities and inconsistencies persist within health systems. The elevated out-of-pocket costs, up to several times the minimum wage, and shortage of resources, makes appropriate diagnosis and treatments inaccessible for many patients living with MG. Novel treatments for MG are unavailable for most people with MG in Latin America. The solution lies not only in increased public spending, but also in structural reform that integrates governance and financing functions, as well as strategic price regulation to reduce the healthcare gap. Implementing a referral program between primary and specialized care could be an option to unlock the management of patients with fewer resources and rare diseases. The global neurology community must come together to help improve care of MG in LAC and other lower income countries. This includes helping train neurologists and neuromuscular specialists who can provide care for patients with MG, building affordable networks for antibody testing, as well as networking with MG specialists around the world to help with challenging cases, when there is scarce local expertise.

## Data Availability

The original contributions presented in the study are included in the article/Supplementary material, further inquiries can be directed to the corresponding author.
