# Supplementary material for: Myasthenia gravis in Latin America and the Caribbean: epidemiology, resources, and accessibility to diagnosis and treatment
Source: Front Public Health. 2026 Apr 8;14:1791605. doi: 10.3389/fpubh.2026.1791605 (PMC13099913; doi:10.3389/fpubh.2026.1791605)
Supplement: Supplementary file 1 [file Table_1.DOCX]

Supplementary Material

**Appendix A. Questionnaire administered to neurologists in Latin America and the Caribbean.**

1. Repetitive nerve stimulation is available in the public and private systems (yes/no).

2. Cost of the repetitive nerve stimulation.

3. Single fiber electromyography is available in the public and private systems (yes/no).

4. Cost of single fiber electromyography.

5. Anti-acetylcholine receptor antibody (AChRA) dosage is available in the public and private systems (yes/no).

6. Cost of AChRA dosage.

7. Muscle-specific anti-tyrosine kinase receptor antibody (anti-MuSK) dosage is available in the public and private systems yes/no).

8. Cost of anti-MuSK dosage.

9. Chest computer tomography (CT) is available in the public and private systems (yes/no).

10. Cost of chest CT.

11. The public system covers pyridostigmine (yes/no).

12. Cost of pyridostigmine 60 mg tablet.

13. The public system covers prednisone (yes/no).

14. Cost of the prednisone 5 mg tablet.

15. The public system covers azathioprine (yes/no).

16. Cost of azathioprine 50 mg tablet.

17. The public system covers mycophenolate 500 mg (yes/no).

18. Cost of mycophenolate 500 mg tablet.

19. The public system covers tacrolimus 1 mg (yes/no).

20. Cost of tacrolimus 1mg tablet.

21. The public system covers Rituximab 500 mg/50ml (yes/no).

22. Cost of rituximab 500 mg/50ml.

23. The public system covers cyclophosphamide 1mg ampoule (yes/no).

24. Cost of cyclophosphamide 1mg ampoule.

25. Immunoglobulins for an exacerbation or crisis are available in the public system. (yes/no).

26. Immunoglobulins as maintenance treatment are available in the public system. (yes/no).

27. Cost of immunoglobulins 1grame.

28. Plasmapheresis is available in the public system (yes/no).

29. Possibility to use eculizumab (yes/no).

30. Additional comments.

# Supplementary Table 1. Ratio between monthly cost of medications if paid out of pocket in relation to the minimum wage.

| **Country** | **Pyridostigmine 60 mg three times per day** | **Prednisone 15 mg per day** | **Azathioprine 100 mg per day** | **Pyridostigmine + Prednisone + Azathioprine** |
| --- | --- | --- | --- | --- |
| Argentina | 0.34 | 0.16 | 0.23 | 0.73 |
| Bolivia | 0.30 | 0.60 | 0.80 | 1.70 |
| Brazil | 0.01 | 0.03 | 0.02 | 0.06 |
| Chile | 0.13 | 0.01 | 0.02 | 0.15 |
| Colombia | 0.73 | 0.20 | 0.12 | 1.05 |
| Ecuador | 0.18 | 0.04 | 0.19 | 0.41 |
| Honduras | 0.43 | 0.03 | 0.23 | 0.67 |
| México | 0.22 | 0.04 | 0.49 | 0.75 |
| Panama | 0.17 | 0.09 | 0.11 | 0.37 |
| Paraguay | 0.13 | 0.05 | 0.13 | 0.31 |
| Uruguay | 0.34 | 0.01 | 0.04 | 0.38 |
| Venezuela | 3.90 | 180.00 | 240.00 | 423.90 |
